# A Versatile Environmental Impedimetric Sensor for Ultrasensitive Determination of Persistent Organic Pollutants (POPs) and Highly Toxic Inorganic Ions

**DOI:** 10.1002/advs.201500013

**Published:** 2015-04-14

**Authors:** Xing Chen, Zheng Guo, Zhong‐Gang Liu, Yu‐Jing Jiang, Dong‐Ping Zhan, Jin‐Huai Liu, Xing‐Jiu Huang

**Affiliations:** ^1^ Nanomaterials and Environment Detection LaboratoryHefei Institutes of Physical ScienceChinese Academy of SciencesHefei230031P. R. China; ^2^ Department of ChemistryUniversity of Science and Technology of ChinaHefei230026P. R. China; ^3^ State Key Laboratory for Physical Chemistry of Solid SurfacesCollege of Chemistry and Chemical EngineeringXiamen UniversityXiamen361005China

**Keywords:** electron‐transfer‐blockage, impedimetric sensor, persistent toxic substances, size matching, ultrasensitivity

## Abstract

An impedimetric sensor for persistent toxic substances, including organic pollutants and toxic inorganic ions is presented. The persistent toxic substances are detected using an ultrasensitive technique that is based on electron‐transfer blockage. This depends on the formation of guest–host complexes, hydrogen bonding, or a cyclodextrin (CD)‐metal complex (M_m_(OH)_n_−β‐CD) structure between the target pollutants and β‐CD.

## 1 Introduction

Electrochemical methods, which can offer excellent merits in terms of high sensitivity, easy‐use, and low cost, have been widely employed in clinical, industrial, environmental, and agricultural applications.^1^ Especially in environmental science, a series of great results have been obtained in the electroanalysis and quantification of toxic micropollutants,^2^ which was achieved mainly based on the redox reactions of target analytes. However, as for such ubiquitous persistent organic ­pollutants (POPs) as polychlorinated biphenyls (PCBs) existing in the environment, their direct electrochemical determination has rarely been reported owing to their chemical inertness and insulating properties,^3^ which was recognized to be the limitation in the development of electrochemical techniques. Besides, in the case of such inorganic ions as Cr(VI), As(III), and As(V), their electrochemical determination was strongly dependent on the using of noble metal nanomaterials and their structures and morphologies of (e.g., gold, silver, and platinum). Gold nanofilm, Au(111)‐like Au electrode, 3D gold nanodendrite network, silver nanoparticles, polymer film platinum nanocomposite, and highly ordered platinum‐nanotube array electrode were therefore designed and fabricated in a complicated way aiming at a more sensitive electroanalysis of such inorganic ions.^4^ Meanwhile, strongly acidic media (e.g., HCl, H_2_SO_4_, HClO_4_, and HNO_3_) were mainly employed as electrolytes,[[qv: 4e]],^5^ which could cause the problems of hydrogen evolution and ­undesirable corrosion. Considering the severe adverse toxic effects of persistent toxic substances (PTS) including POPs and highly toxic inorganic ions in environment and human health, such as genotoxicity, tumor promotion, lung cancer, skin allergy, and arsenicosis,^6^ it is critically challenging and necessary to explore the novel and simple electrochemical method on their determination.

Alternatively, electrochemical impedance spectroscopy (EIS) is currently attracting a great deal of attention, which is a powerful and efficient tool for analyzing the electrode‐solution interface and sensitively detecting that change in complex electrical resistance.^7^ Recently, EIS technique has been developed for studying the fundamental and applied electrochemistry and materials science, including characterization of materials,^8^ biosensors in sensing of bacterial, DNA or protein,^9^ corrosion science,^10^ fuel cell, and batteries.^11^ Despite these abroad applications, only a few studies have reported on the electrochemical detection of POPs at μm level by our group,^12^ which could not satisfy the demand of environmental science. In combination with the excellent properties of microelectrode and nanoelectrode, we expect that the ultrasensitive analysis of POPs and highly toxic inorganic ions could be achieved based on EIS technique. To the best of our knowledge, such interesting and meaningful work has not been developed.

Herein, we demonstrate an impedimetric sensor for PTS determination by introducing mercapto‐β‐cyclodextrin (β‐CD) self‐assembly monolayers (SAMs) onto 2 mm, 25 μm, and 400 nm diameter gold electrodes. Cyclodextrins (CDs) explored as molecular hosts are capable of including small hydrophobic molecules inside their cavities in aqueous media.^13^ The sensing mechanism is based on the formation of guest–host complexes or the hydrogen bonding between Cr(VI), As(III), and As(V), and β‐CD under neutral conditions (pH 7.4), as well as the formation or dissociation of M*_m_*(OH)*_n_*−β‐CD complexes (M: Cu(II), Zn(II), Cd(II), Pb(II), and Mn(II)) through a reversible pH‐stimulation. The complexes cause the specific inhibition of electron transfer of the redox probe, Fe(CN)_6_^3−/4−^, in the sensing interface, leading a change in electron‐transfer resistance before and after sampling. The results suggest that such a simple and smart platform with mercapto‐β‐CD modified gold microelectrode is expected to be useful in the impedance analysis of PTS (including POPs and highly toxic inorganic ions) with ultrasensitivity.

## 2 Results and Discussion

**Figure 1:**
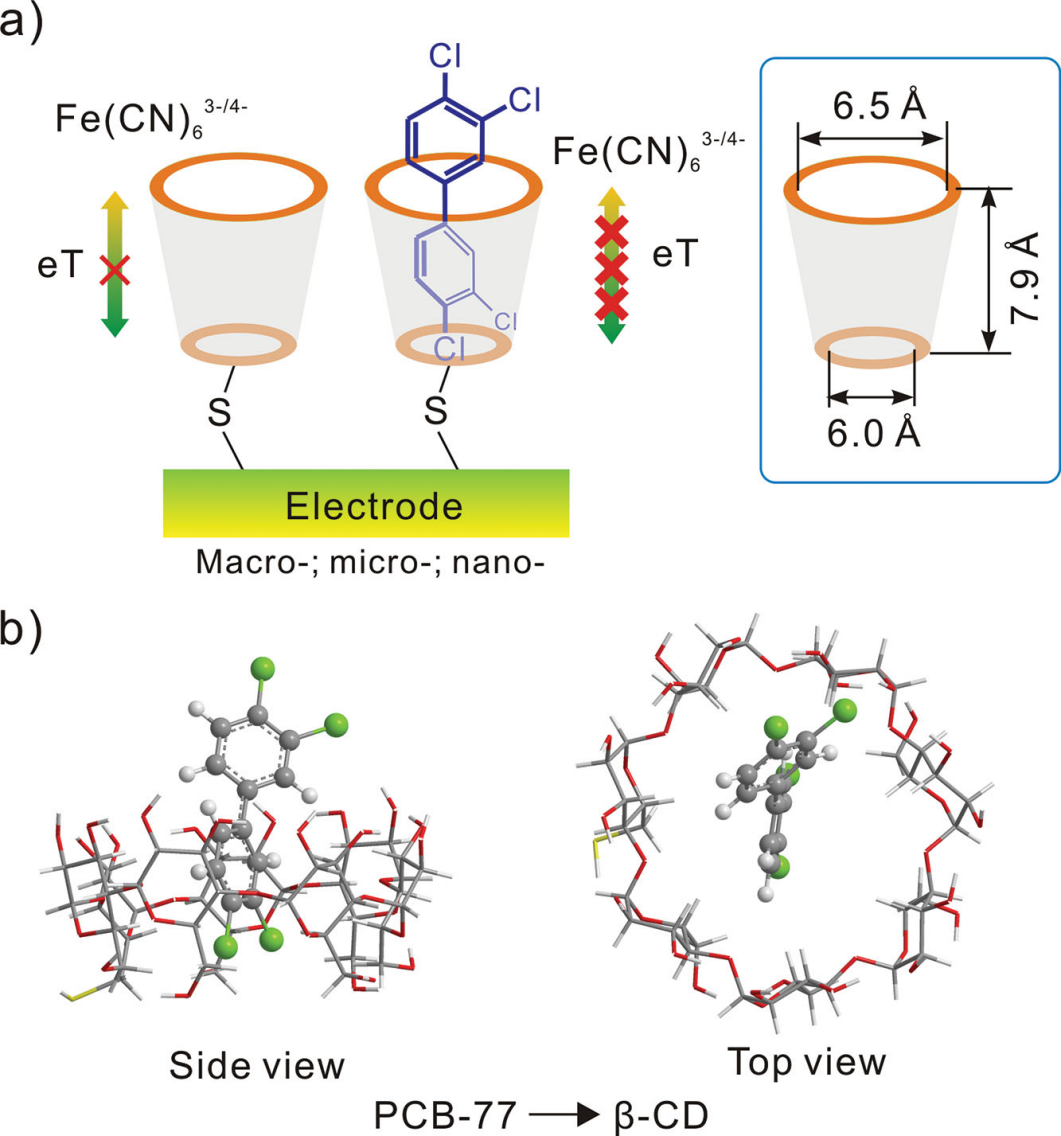
a) Idealized view of mercapto‐β‐CD‐decorated macro, micro, and nanogold electrodes before and after their interaction with POPs. Changes in the electron‐transfer resistance before and after sampling, expressed as a relative increase, and gave a quantitative amount of POPs. The more increase in electron‐transfer resistance occurred, the more amount of POPs would be given. b) How β‐CDs interact with guest molecules (POPs) when they are being sensed in a solution.

**Figure** **1** briefly illustrates our strategy, in which an impedimetric sensor was fabricated for the determination of POPs that generally based on electron‐transfer‐blockage. Herein mercapto‐β‐cyclodextrin (β‐CD) monolayer was self‐assembled via Au–S bond onto macro, micro, and nanogold electrodes (that is, 2 mm, 25 μm, and 400 nm diameter, Figure 1a). Cyclodextrin is characterized as a conical cylinder with a hydrophobic inner cavity and a hydrophilic exterior.^14^ As depicted in Figure 1b, a PCB‐77 molecule, as a representative in POPs, with appropriate size can serve as a guest molecule and be captured into the internal cavity of β‐CDs based on the hydrophobic interactions, thus forming stable host–guest inclusion complexes.[[qv: 13c]],^15^ Because of the insulating property of PCB‐77, an electrical barrier was constructed on the surface of electrode, which further increased the electron‐transfer‐blockage in this system. Changes in the electron‐transfer resistance before and after sampling gave a quantitative amount of POPs. The more increase in electron‐transfer resistance occurred, the more amount of POPs would be given, which can be used for detection and quantification of POPs.

We first characterized the different dimension of working electrodes (Figure S1, Supporting Information). Due to the different dimension of working gold electrodes, different cyclic voltammograms (CVs) and Nyquist diagrams of EIS were presented. As the electrode dimension reduced, the absolute current decreased while the corresponding resistance increased, which was attributed to the differential mass diffusion profiles.^16^ After modification with mercapto β‐CD, it can be observed that the electron‐transfer resistance (*R*_et,mod_, semicircle domain in EIS) increased by different degrees in comparison with the 2 mm, 25 μm, and 400 nm diameter bare gold electrode, indicating that mercapto‐β‐CD SAMs had been decorated onto the working electrode surface. Assuming that when the mercapto‐β‐CD was 100% covered onto the bare electrode the *R*_et,_ _mod_ was much higher than the *R*_et,_ _bare_. The *R*_et_ in EIS for three‐kind of electrodes before and after mercapto‐β‐CD modification were calibrated (Figure S2, Supporting Information). The electrode surface coverage *θ* modified with mercapto‐β‐CD SAMs was calculated as 0.921 ± 0.008, 0.807 ± 0.029, and 0.388 ± 0.039 for 2 mm, 25 μm, and 400 nm diameter gold ­electrodes, respectively.

**Figure 2:**
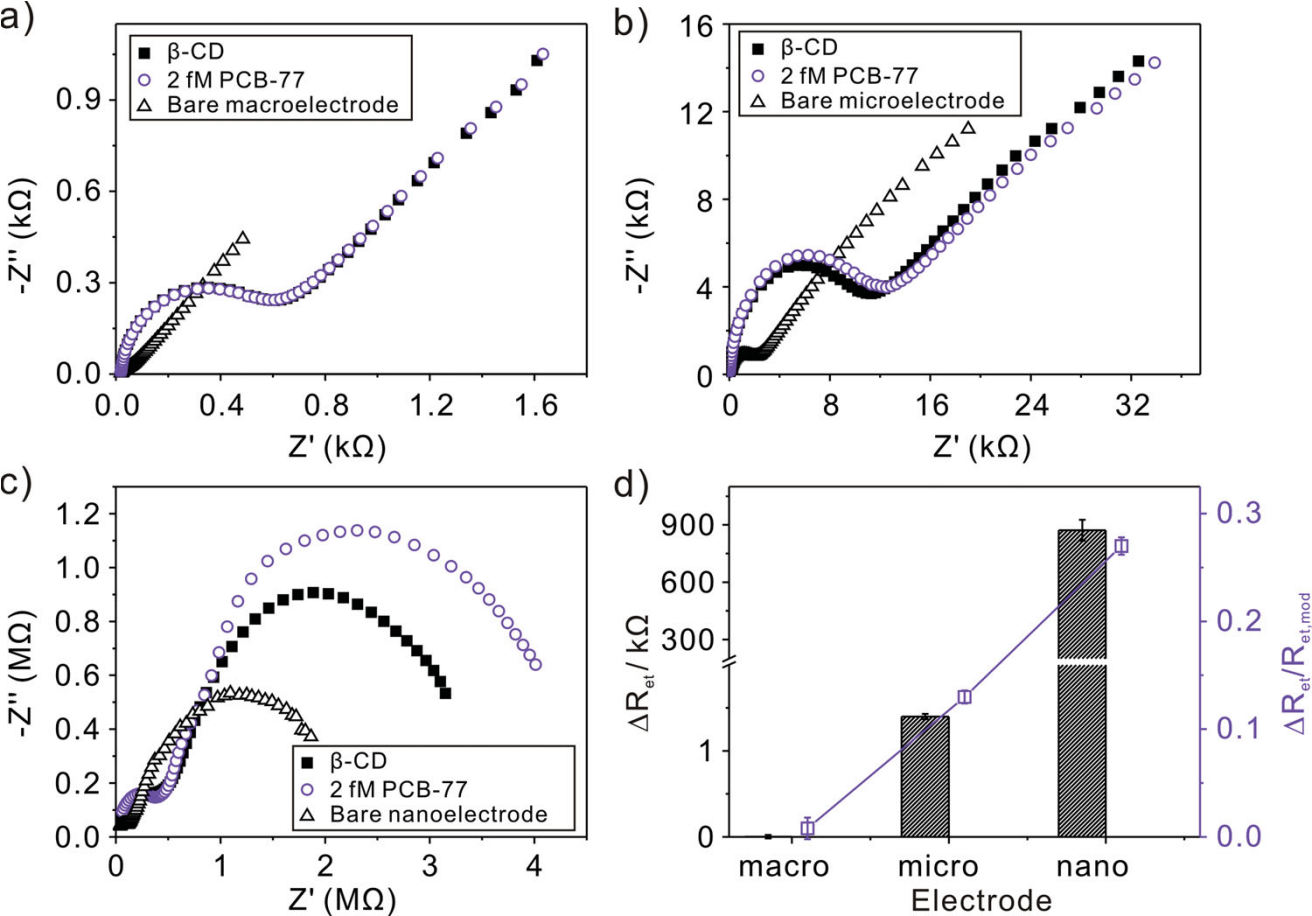
Nyquist diagram of EIS responses at mercapto‐β‐CD modified a) 2 mm, b) 25 μm, and c) 400 nm diameter gold electrodes before and after preconcentration of 2 × 10^−15^ m PCB‐77 for 1 h. d) Comparison of *R*_et_ change and its increasing rate (Δ*R*_et_/*R*_et,mod_) on three kinds of electrode. Data were collected from panels (a‐c).

Subsequently, the efficiency of mercapto‐β‐CD SAMs modified 2 mm, 25 μm, and 400 nm diameter gold electrodes toward POPs was tested. With immersion into the solutions containing 2 × 10^−15^ m PCB‐77 for 1 h, their impedance behaviors toward ultratrace PCB‐77 were presented in **Figure** **2**. No change in the EIS response was observed at mercapto‐β‐CD 2‐mm‐diameter electrode before and after interacting with PCB‐77 (Figure 2a), revealing the weak resolution of the modified electrode toward PCB‐77. With reducing the dimension of the electrode, *R*_et_ increased and the resolution toward PCB‐77 was more obvious (Figure 2b,c), where the resolution was defined as Δ*R*_et_/*R*_et,mod_ (Δ*R*_et_ = *R*_et,PCB_ – *R*_et,mod_). The discernible Δ*R*_et_ of 1.43 and 885 kΩ, as well as the resolution of Δ*R*_et_/*R*_et,mod_ ratio of 0.134 and 0.272, toward PCB‐77 were observed at mercapto‐β‐CD modified 25 μm and 400 nm diameter electrode, respectively, which were more apparent than that at the modified 2 mm diameter electrode (Δ*R*_et_: 5Ω, Δ*R*_et_/*R*_et,mod_: 0.008) (Figure 2d). It was worth pointing out that the modified micro and nanoelectrode possessed the effective capture capacity and high resolution in the analysis of the ultratrace target molecules PCB‐77.

**Figure 3:**
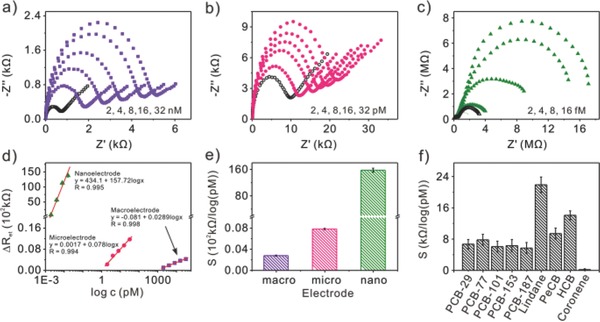
Nyquist diagram of EIS responses at mercapto‐β‐CD modified a) 2 mm, b) 25 μm, and c) 400 nm diameter gold electrode with different concentrations of PCB‐77 in PBS saline solution containing 0.1 m KCl and 5 × 10^−3^ m Fe(CN)_6_^3‐/4−^, respectively. d) Calibration plots of Δ*R*_et_ against the logarithmic value of PCB‐77 in different concentration ranges. Data were collected from panels (a–c). e) Sensitivity (Δ*R*_et_/log*c*) comparison of mercapto‐β‐CD modified 2 mm, 25 μm, and 400 nm diameter gold electrodes toward PCB‐77. f) Detection of nine POP molecules at mercapto‐β‐CD modified 25 μm diameter gold electrode. Data were collected from corresponding electrochemical impedance spectra (Figure S4, Supporting Information).

Furthermore, we explored the impedance behaviors of mercapto‐β‐CD modified 2 mm, 25 μm, and 400 nm dia­meter gold electrodes toward PCB‐77 over a concentration range (**Figure** **3**a–c and optimized condition of preconcentration time in Figure S3, Supporting Information). The electron‐transfer resistances were found to continually increase at the three kind of mercapto‐β‐CD modified gold electrode as the PCB‐77 concentrations were increased. Interestingly, it should be noted that with the reduction in dimension of the modified electrode, the limit of quantitation was decreased, that is, the determination of PCB‐77 can be realized varying from nm to fm level with the electrode down to nanoscale. Figure 3d gives the linear relationship between Δ*R*_et_ and the logarithmic value of PCB‐77 concentration at mercapto‐β‐CD modified 2 mm, 25 μm, and 400 nm diameter gold electrodes over a different concentration range. The obtained sensitivities (Δ*R*_et_/log*c*) were 2.89 kΩ per log nm, 7.80 kΩ per log pm, and 15.7 MΩ per log pm at modified macroelectrode, microelectrode, nanoelectrode, respectively (Figure 3e). The limits of detection (a signal‐to‐noise ratio (S/N) of 3) of 4 × 10^−12^ m, 0.249 × 10^−12^ m, and 0.209 × 10^−15^ m were correspondingly achieved. The amazing results suggested that the ultrasensitive determination of PCB‐77 can be implemented with the electrochemical impedance technique based on electron‐transfer blockage. The reduction in dimension of modified electrode led to the higher sensitivity and lower detection limit, which was attributed to the different diffusion models.^17^ Especially, in comparison with the modified 2 mm gold electrode, mercapto‐β‐CD modified 25 μm, and 400 nm diameter gold electrodes can be applied in the determination of ultratrace PCB‐77 at pm and fm level.

In the case of the modified 400‐nm‐diameter gold electrode, although ultrahigh resolution toward PCB‐77 can be obtained, it inevitably suffered from the complicated fabrication procedures and sophisticated instrument. Furthermore, referring to the self‐assemble process, the surface coverage θ of modified nanoelectrode (0.388 ± 0.039) was far less than that modified microelectrode (0.807 ± 0.029). Remarkably, the modified microelectrode fabricated in a simple way was good enough to develop in analysis of ultratrace PCB‐77 with high sensitivity. Next, we evaluate the efficiency of mercapto‐β‐CD modified 25 μm diameter gold electrode in the determination of POPs, nine POPs with different sizes, namely, PCB‐29, PCB‐77, ­PCB‐101, PCB‐153, PCB‐187, lindane, pentachlorobenzene (PeCB), hexachlorobenzene (HCB), and coronene, were further investigated in a phosphate‐buffered saline (PBS) solution (pH 7.4). EIS responses and corresponding calibration plots toward the target molecules were shown in Figure S4, Supporting Information. Figure 3f presents a comparison of sensitivities for individual analysis of these target molecules. Clearly, there were the similar sensitivities toward the four PCBs, which were slightly lower than that of chlorobenzenes (PeCB and HCB) and lindane. Moreover, in comparison with previous reports, this platform realized the detection of PCBs and chlorobenzenes with the highest sensitivity and lowest limit of detecition (LOD).^18^ However, in the analysis of coronene that was a relatively larger molecule, no obvious change in *R*_et_ as well as a negligible sensitivity were observed even with the addition of coronene up to 20 000 × 10^−12^ m. We suggest that the different impedimetric sensing may be attributed to the size matching effect between host and gust molecules.^19^

**Figure 4:**
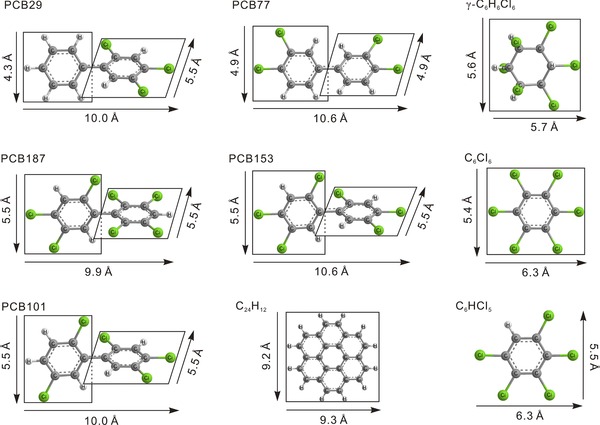
Molecular models and calculated dimensions of POPs predicted by DFT method using Gaussian 03 software package.

A scientific understanding on the different impedimetric sensing was further demonstrated. As known, β‐CD possesses the special properties and unique structure of a rather rigid hydrophobic cylinder with cavity depth about 7.9 Å and diameter about 6.0–6.5 Å (Figure 1a),[[qv: 13a]] which was quite suitable to serve as molecular receptor and tended to interact with some guest molecules. Generally, the principle of size matching was one of important factors in the formation of host–guest inclusion complexes,^20^ which was of benefit for proceeding with the examination of our results. In combination with DFT method, the 1D parameters of the target molecules were calculated and **Figure** **4** illustrates the corresponding information in detail. Two of 1D sizes of PCB‐29 (4.3 Å, 5.5 Å), PCB‐77 (4.9 Å, 4.9 Å), PCB‐101 (5.5 Å, 5.5 Å), PCB‐153 (5.5 Å, 5.5 Å), and PCB‐187 (5.5 Å, 5.5 Å) were smaller than the diameter of β‐CD. Therefore, these five guest molecules can easily enter the cavity of β‐CD to form inclusion complexes, which made it possible to block the electron transfer and accordingly increase the electrochemical resistance, *R*_et_. In the case of target molecules with planar structure, dimensional parameters of PeCB (5.5 Å, 6.3 Å) and HCB (5.4 Å, 6.3 Å) and lindane (5.6 Å, 5.7 Å) were all smaller than that of β‐CD, which increased the possibility of target molecules to enter into the cavity of β‐CD. Relatively better sensitivities were achieved, especially toward lindane. As for coronene, its dimension sizes (9.2 and 9.3 Å) were larger than that of β‐CD, leading a difficulty for coronene to enter into the cavity of β‐CD. Thus the electron‐transfer resistance, *R*_et_, was almost similar before and after preconcentration of coronene over a concentration range (Figure S4, Supporting Information). Based on the results, it was clear that the above analysis was in good agreement with our experimental data. Notably, herein β‐CD strictly performed the rule of size matching and possessed the selectivity to form the host–guest inclusion complexes during the molecular process.[[qv: 13c]],[[qv: 19a]] Depending on the size matching effect between β‐CD and POPs, it may be difficult to discriminate the POPs molecules from another in this class because of the similar sizes. Thus, the impedimetric sensor was suggested to be capable to quantify total concentrations of all PCBs with this size range.[[qv: 19a]],^21^

**Figure 5:**
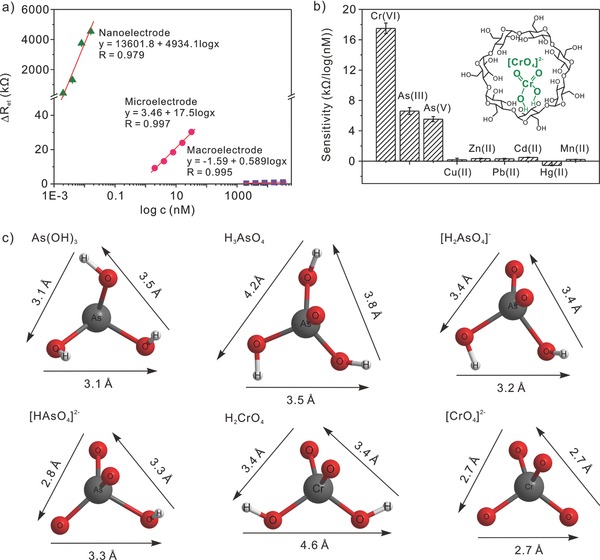
a) Calibration plots of Δ*R*_et_ against the logarithmic value of Cr(VI) concentrations on mercapto‐β‐CD modified 2 mm, 25 μm, and 400 nm diameter gold electrode. Data were collected from electrochemical impendence spectra. b) Comparison of sensitivity of Cr(VI), As(III), and As(V) in neutral solution on mercapto‐β‐CD modified 25 μm diameter gold electrode. Data were collected from corresponding EIS responses (Figure 5a and Figure S5, Supporting Information). Inset: the interaction between β‐CD and CrO_4_^2−^ through H‐bond. c) Molecular models of Cr(VI), As(III), and As(V) in neutral solution and calculated dimensions. The structures were predicted by DFT method using Gaussian 03 software package.

Since the impedimetric sensing of POPs was developed, we found that the sensing performance toward Cr(VI), As(III), and As(V) can be realized in different concentration ranges. Taking Cr(VI) sensing as an example, a sensitivity of 4934.1 kΩ per log nm over concentration range 2 × 10^−12^ m to 32 × 10^−12^ m was obtained on mercapto‐β‐CD modified nanoelectrode. Whereas the response range of microelectrode and macroelectrode was in nm and μm, respectively. The sensitivities on micro and nanoelectrode were almost 300 times and 8000 times higher than that on modified macroelectrode, respectively (**Figure** **5**a). Based on the same consideration, a mercapto‐β‐CD modified 25 μm diameter gold was selected for the investigation of the representative inorganic ions (e.g., As(III), As(V), Cu(II), Zn(II), Hg(II), Cd(II), Pb(II), and Mn(II)) (see Figure S5, Supporting Information for EIS data). Figure 5b gives a comparison of the sensitivities of these target ions at mercapto‐β‐CD modified 25 μm diameter gold electrode, which was derived from three independent experiments. As depicted, the sensitivities for Cr(VI), As(III), As(V) were much higher than that of Cu(II), Zn(II), Hg(II), Cd(II), Pb(II), and Mn(II), which was recognized as the remarkable interaction between Cr(VI), As(III), As(V), and β‐CD.^22^ The impedimetric sensing performance toward As(III) (sensitivity: 7.24 kΩ per log nm; LOD: 0.26 × 10^−9^ m) and Cr(VI) (sensitivity: 17.5 kΩ per log nM; LOD: 0.24 × 10^−9^ m) under mild and neutral conditions was much better than those based on electrochemical redox reaction under strong acidic or harsh conditions (Tables S1 and S2, Supporting Information). Inset in Figure 5b illustrates the interaction between CrO_4_^2−^ and β‐CD via hydrogen bonding. It has been reported that different species of these high valence ions were existed in neutral solution: CrO_4_^2−^ for Cr(VI), As(OH)_3_ for As(III) and HAsO_3_^2−^, and AsO_4_^3−^ for As(V).[[qv: 13c]],^23^ The visual molecular models and calculated dimensions of Cr(VI), As(III), and As(V) in neutral solution are shown in Figure 5c. The presence of Cr(VI), As(III), and As(V) in the cavity of β‐CD would hinder the electrochemical process of redox probe, Fe(CN)_6_^3−/4−^, thus leading to the increase in the electron‐transfer resistance, *R*_et_. Besides, no obvious inference can be observed in the presence of such high‐valence inorganic ions as Fe(III), Al(III), and Co(III) because of the occurrence of precipitation under pH 7.4 (*p*K_sp_ for Fe(OH)_3_ Al(OH)_3_, Co(OH)_3_: 37.40, 32.34, 43.80).^24^

On the basis of the above results, it was recognized that our strategy, the impedimetric sensor can be efficiently developed for the ultrasensitive determination of such highly toxic ions. However, in respect to such bivalent metal ions as Cu(II), Zn(II), Cd(II), Pb(II), and Mn(II), their predominant species mainly existed as M^2+^ in neutral aqueous solution at low concentration. Because of lack of the appropriate binding site, such free ion, M^2+^, could not interact with β‐CD. Thus even the concentration of Cu(II), Zn(II), Cd(II), Pb(II), and Mn(II) up to 20 × 10^−6^ m, no change in the electron‐transfer resistance, *R*_et_, was observed (Figure S5, Supporting Information).

**Figure 6:**
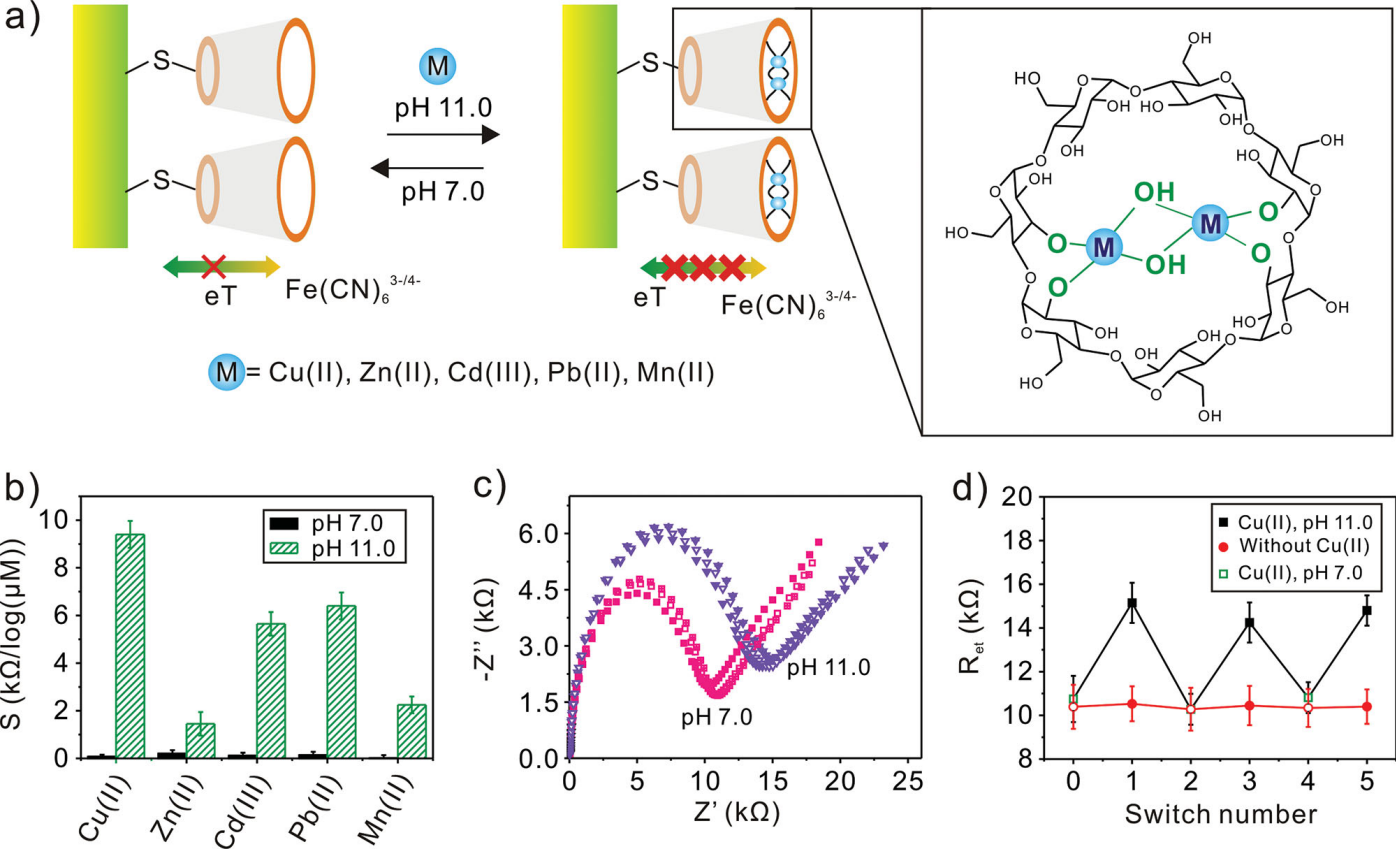
a) pH‐switchable control of electron transfer on a mercapto‐β‐CD modified gold electrode through the reversible pH‐stimulated formation and dissociation of a CD‐metal complexes (M_2_(OH)_2_β‐CD^2−^) structure. b) A comparison in sensitivity (Δ*R*_et_/log*c*) of mercapto‐β‐CD modified 25 μm diameter gold electrode toward Cu(II), Zn(II), Cd(II), Pb(II), Mn(II) with preconcentration of 1 h in solution of pH 7.4 and pH 11.0. c) Nyquist diagram of EIS responses toward 1 × 10^−6^ m Cu(II) at mercapto‐β‐CD modified 25 μm diameter gold electrode after preconcentration for 1 h. The preconcentration process is alternately cycled between pH 7.4 and 11.0. d) Oscillation behavior of electron‐transfer resistance, *R*_et_, between pH 7.4 and 11.0. Data were collected from (c). Red line corresponds to the control experiments that performed after a preconcentration at pH 11.0 in the absence of Cu(II).

As mentioned, the interaction of β‐CD with such bivalent metal ions was generally weak in acidic and neutral solutions. However, at alkaline media, the case would be quite different since deprotonated hydroxyl groups (^−^O‐groups) in β‐CD can serve as receptors for metal ions.[[qv: 15a]],^25^ This has provoked research interest to evaluate the impedimetric sensing performance at mercapto‐β‐CD modified 25 μm diameter gold electrode toward such bivalent metal ions at alkaline solutions. **Figure** **6**a schematically outlines the pH‐switchable control of electron transfer resistance at a mercapto‐β‐CD modified gold electrode, in which *R*_et_ was reversibly switched by cycling the pH of the solution between high and low values, respectively.

The impedimetric sensing toward Cu(II), Zn(II), Cd(II), Pb(II), and Mn(II) over a concentration range was implemented, respectively, after a preconcentration at alkaline solution (pH 11.0) (Figure S6, Supporting Information). The corresponding sensitivities were summarized in Figure 6b. It can be observed that the obtained sensitivities toward these metal ions were significantly higher than that obtained in neutral solution (pH 7.4). We suggest that the differences in sensitivities were attributed to the different binding interaction of the metal ions with β‐CD. As reported, in alkaline solution, the deprotonated 2‐ and 3‐OH of β‐CD can coordinate with Cu(II) to form Cu_2_(OH)_2_β‐CD^2−^ complexes through a binuclear hydroxy bridge structure.^26^ The binding molar ratio of β‐CD with Cu(II) was 1:2. Besides, the inorganic ions Pb(II), Cd(II), and Mn(II) can respectively bind with β‐CD to form such complexes as Pb(II)β‐CD, Cd(OH)_2_β‐CD^2−^, and Mn_2_(OH)_2_β‐CD^2‐^ at alkaline solution in molar ratios of 1:1, 1:1, and 1:2.[[qv: 15a]],^27^ The existence of M*_m_*(OH)*_n_*−β‐CD complexes on the surface of modified gold electrode could hinder the redox process of probe, Fe(CN)_6_^3−/4−^, resulting in the increase in *R*_et_ (Figure 6a and Figure S6, Supporting Information).

Figure 6c shows the repetitive impedimetric sensing of 1 × 10^−6^ m Cu(II) at mercapto‐β‐CD modified 25 μm diameter gold electrode with cycling the preconcentration process between pH 7.4 and 11.0. A reversible switch of EIS was clear between pH 7.4 and pH 11.0. A lower electron‐transfer resistance (*R*_et_, ≈10.87 kΩ) was presented at pH 7.4, which was very similar to that of freshly prepared modified electrode, showing that no interaction between β‐CD and Cu(II) occurred at pH 7.4. Whereas *R*_et_ increased to about 15.03 kΩ at pH 11.0, which was due to the formation of Cu_2_(OH)_2_β‐CD^2−^ complexes. As modulating pH to 7.4, *R*_et_ was almost returned to the initial state because of the dissociation of Cu_2_(OH)_2_β‐CD^2−^ structure. The *R*_et_ reversible change between 7.4 and 11.0 was summarized in Figure 6d. As a comparison, no change in *R*_et_ was found with cycling pH between 7.4 and 11.0 in the absence of Cu(II).

To get more details on the effect of pH, we investigated the impedimetric sensing toward the metal ions over a different pH value ranging from 7.4 to 11.0 by taking Pb(II) as an example. The obtained sensitivities were presented in Figure S7, Supporting Information, in which the continually improved sensitivity toward Pb(II) was observed. The interesting results further confirmed that ^−^O‐groups of β‐CD could efficiently bind with ions and there would be more ^−^O‐groups participating the formation of M*_m_*(OH)*_n_*−β‐CD complexes with increasing pH values. Importantly, the high sensitivities obtained from our strategy toward small metal ions in alkaline solution could give the great promise for application in the alkaline sample.

It is worth pointing out that the mercapto‐β‐CD modified 25 μm diameter gold electrode exhibited robust stability in neutral and alkaline conditions (pH < 11) (Figure S8, Supporting Information), which was of benefit for the practical application in environmental science. However, when pH value exceeded 11, the instability of the modified microelectrode would be observed and the surface coverage was decreased (Figure S9, Supporting Information). It suggested that the thiolated SAMs chemisorpted on gold electrode surface may undergo desorption by breaking Au–S bond in strongly alkaline solutions. Besides, the mercapto‐β‐CD modified gold microelectrode was unsuitable in the electroanalysis of Hg(II). Its addition in solution would lead to a remarkable decrease in *R*_et_, as well as the surface coverage *θ*, of the modified electrode (Figure S10, Supporting Information). This was attributed to the decomposition of Au–S bond and competitive formation of Hg−S bond, leading to the instability of the mercapto‐β‐CD modified gold electrode.

This is an open access article under the terms of the Creative Commons Attribution License, which permits use, distribution and reproduction in any medium, provided the original work is properly cited.

## 3 Conclusion

In summary, we successfully fabricated an impedimetric sensor for ultrasensitive determination of PTS by introducing mercapto‐β‐CD SAMs onto macro, micro, and nano gold electrodes. The different impedance behaviors of the modified electrode resulting from different dimension sizes were demonstrated. Based on the formation of guest–host complexes or the intermolecular hydrogen bonding between Cr(VI), As(III), As(V), and β‐CD, the mercapto‐β‐CD modified microgold electrode was implemented in the ultrasensitive determination of POPs at pm level and highly toxic inorganic ions (Cr(VI), As(III), and As(V)) at nm level under mild and neutral conditions (pH 7.4). In addition, based on the strategy, such heavy metal ions as Cu(II), Zn(II), Cd(II), Pb(II), and Mn(II) were successfully analyzed with regulating the suitable pH value, depending on the formation or dissociation of M*_m_*(OH)*_n_*−β‐CD complexes. The simple and smart platform can be contributed to giving the efficient settlement in the successful determination of nonelectroactive and persistent toxic substances at ultratrace concentrations and remarkably extending the application spectrum of electrochemical methods. Moreover, much more work could be further developed to improve the discrimination in the multiple analytes and to provide a deep and scientific explanation on the interaction between β‐CD and analytes.

## 4 Experimental Section

*Materials*: All the chemicals were of analytical grade and used as purchased without further purification. γ‐hexachlorocyclohexane (lindane), coronene, and five kinds of PCBs named 2,4,5‐trichlorobiphenyl (PCB‐29), 3,3′,4,4′‐tetrachlorobiphnyl (PCB‐77), 2,2′,4,5,5′‐pentachlorobiphenyl (PCB‐101), 2,2′,4,4′,5,5′‐hexachlorobiphenyl (PCB‐153), and 2,2′,3,4′,5,5′,6‐heptachlorobiphenyl (PCB‐187) were obtained from J&K Chemical Ltd., Shanghai. Hexachlorobenzene (HCB), pentachlorobenzene (PeCB), and other inorganic reagents were received from Sinopharm Chemical Reagent Co., Ltd., China. A phosphate‐buffered saline solution (PBS, pH 7.4) of 0.1 m was prepared by dissolving 1.6 g of KCl, 64 g of NaCl, 1.92 g of KH_2_PO_4_, and 11.52 g of K_2_HPO_4_ in 800 mL in ultrapure fresh water. Standard stock solutions of POPs (100 × 10^−9^ m) were prepared in anhydrous ethanol. Ultrapure fresh water (specific resistivity >18 MΩ cm) was used throughout the experiments obtained from a Millipore water purification system (MilliQ, S.A., Molsheim, France).

*Decoration of Mercapto‐β‐CD*: The fabrication of the self‐assembly monolayer (SAM) of mercapto‐β‐CD onto gold macro, micro, and nanoelectrode was carried out by immersing the freshly prepared gold electrode into a saturated aqueous solution of mercapto‐β‐CD for 12 h (macroelectrode), 1 h (microelectrode), and 0.5 h (nanoelectrode) at room temperature and then mercapto‐β‐CDs were decorated onto the Au electrode surface via the Au−S bond.

*Electrochemical Measurements*: All electrochemical measurements were performed using a CHI 660D computer‐controlled potentiostat (ChenHua Instruments Co., Shanghai, China) with a standard three‐electrode configuration, which employed mercapto‐β‐CD modified 2 mm, 25 μm, and 400 nm diameter gold electrodes as the working electrodes, a Ag/AgCl electrode as the reference electrode and a platinum wire (1 mm in diameter) as the counter electrode. The characterization of mercapto‐β‐CD modified 2 mm, 25 μm, and 400 nm diameter gold electrodes was carried out with cyclic voltammetry (CV) and electrochemical impedance spectroscopy (EIS). The impedimetric sensing of persistent toxic substances (PTS, including POPs and inorganic ions) was performed in a PBS saline solution (pH 7.4) containing 5 × 10^−3^ m Fe(CN)_6_^3−/4−^ and 0.1 m KCl, followed by a preconcentration of target molecules for 1 h over a concentration range in water solution (pH 7.0) or NaOH solution (pH > 7.0).

## Supporting information

As a service to our authors and readers, this journal provides supporting information supplied by the authors. Such materials are peer reviewed and may be re‐organized for online delivery, but are not copy‐edited or typeset. Technical support issues arising from supporting information (other than missing files) should be addressed to the authors.

Supplementary
