# Supplementary material for: A Versatile Environmental Impedimetric Sensor for Ultrasensitive Determination of Persistent Organic Pollutants (POPs) and Highly Toxic Inorganic Ions
Source: Adv Sci (Weinh). 2015 Apr 14;2(5):1500013. doi: 10.1002/advs.201500013 (PMC5115365; doi:10.1002/advs.201500013)
Supplement: Supplementary file 1 — Supplementary [file ADVS-2-0i-s001.pdf]

Copyright WILEY-VCH Verlag GmbH & Co. KGaA, 69469 Weinheim, Germany, 2015.

ADVANCED SCIENCE
Open Access

Supporting Information

for *Adv. Sci.*, DOI: 10.1002/advs. 201500013

A Versatile Environmental Impedimetric Sensor for Ultrasensitive Determination of Persistent Organic Pollutants (POPs) and Highly Toxic Inorganic Ions

*Xing Chen, Zheng Guo, Zhong-Gang Liu, Yu-Jing Jiang, Dong-Ping Zhan, Jin-Huai Liu, and Xing-Jiu Huang**

Copyright WILEY-VCH Verlag GmbH & Co. KGaA, 69469 Weinheim, Germany, 2013.

Supporting Information

A Versatile Environmental Impedimetric Sensor for Ultrasensitive Determination of Persistent Organic Pollutants (POPs) and Highly Toxic Inorganic Ions

*Xing Chen[1], Zheng Guo[1], Zhong-Gang Liu[1,2], Yu-Jing Jiang[1,2], Dong-Ping Zhan[3], Jin-Huai Liu[1] and Xing-Jiu Huang[1,2,]**

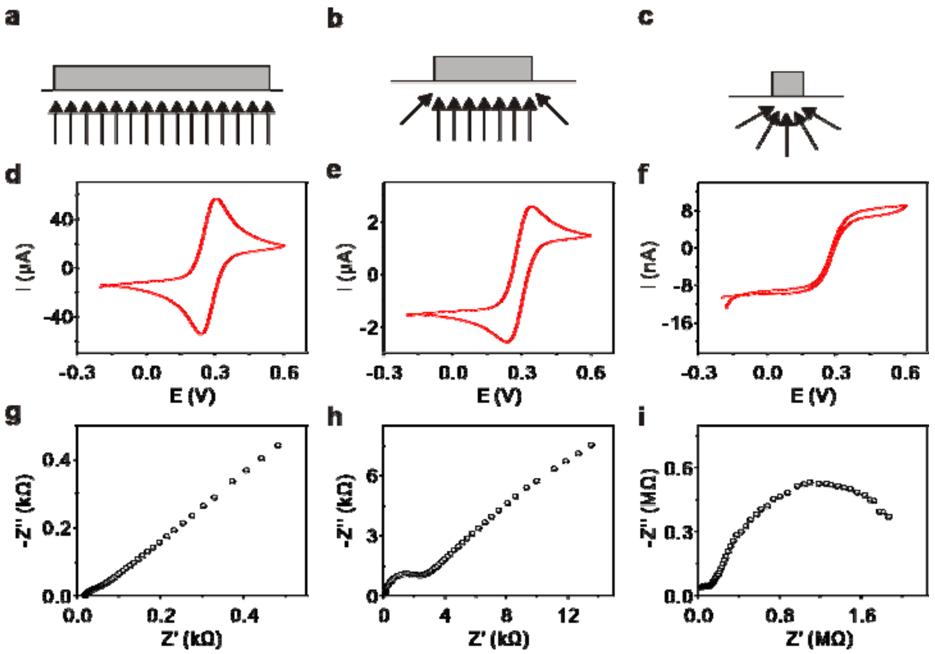

Figure S1. A comparison of electrochemical behavior with electrode dimension reduction. a,b,c) Diffusion models, d,e,f) cyclic voltammograms, and g,h,i) Nyquist diagrams of EIS for 2-mm, 25-μm, and 400-nm-diameter gold electrode in PBS saline solution containing 5 mM $Fe(CN)_6^{3-/4-}$. In d,e,f), scan rates are 0.1 V s^{-1} for macro- and microelectrode, and 0.01 V s^{-1} for nanoelectrode. In g,h,i)**,** frequency ranges from 1 Hz to 100000 Hz for macro- and microelectrode; and 0.1 Hz to 10000 Hz for nanoelectrode are applied with a signal amplitude of 5 mV).

It was extremely different from the diffusion models on the electrode with different dimension sizes. Figure S1a-c give the changes in mass transport from one-dimensional linear diffusion for conventional macroelectrode (2-mm-diameter gold electrode) to multi-dimensional radial diffusion for nanoelectrode (400-nm-diameter gold electrode).[1] Meanwhile, linear diffusion may result in a classical CV curve, and radial diffusion leads to a steady-state and attains an ideal sigmoidal shape,[2] just as shown in Figure S1d-e. In detail, obvious reverse peak currents were observed for macroelectrode and nearly sigmoidal shape

with small reverse appeared for microelectrode (25-μm-diameter gold electrode), whereas an ideal sigmoidal shape was presented at nanoelectrode. The reduction in dimension of electrode from macro- to nano- sizes resulted in the decrease in the current by almost four orders of magnitude. This would cause a corresponding increase in the electrochemical resistance (Figure S1g-i).

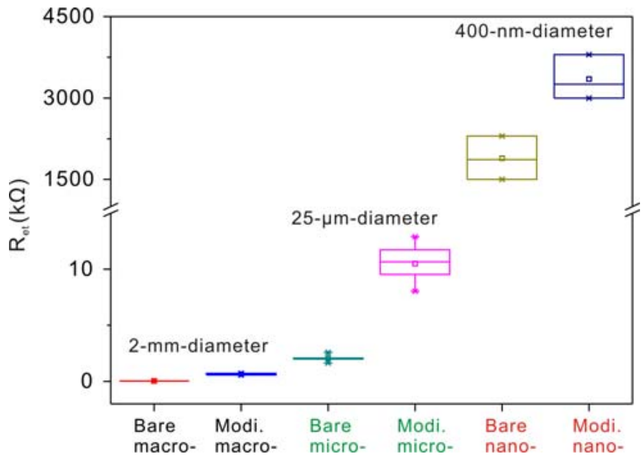

Figure S2. Change in EIS respones before and after modification at mercapto-β-CD modified 2-mm, 25-μm, and 400-nm-diameter gold electrode in PBS saline solution of 5 mM $Fe(CN)_6^{3-/4-}$ and 0.1 M KCl.

The impedance behaviors of 2-mm, 25-μm, and 400-nm-diameter gold electrode before and after modification with mercapto-β-CD SAMs were characterized with electrochemical impedance spectroscopy. As shown in Figure S2, more obvious changes on the electron-transfer resistance, R_{et}, can be observed with the reduction in electrode dimensions. Moreover, based on the changes in R_{et} before and after modification, the surface coverage θ can be calculated by the following equation:[3]

$$\theta = 1 - \frac{R_{et,bare}}{R_{et,\mathrm{mod}.}}$$

where $R_{et,bare}$ represented the electron-transfer resistance of bare gold electrode, $R_{et,\mathrm{mod}.}$ was the corresponding resistance of mercapto-β-CD SAMs modified gold electrode. Herein assuming that when the mercapto-β-CD was 100% covered onto the bare electrode the $R_{et,\mathrm{mod}.}$

was much higher than the $R_{et,bare}$. The surface coverages θ were therefore achieved as 0.921 ± 0.008 for macroelectrode, 0.807 ± 0.029 for microelectrode, and 0.388 ± 0.039 for nanoelectrode. Each value was expressed as mean ± standard deviation (n = 3). The surface coverage θ of modified nanoelectrode was far less than that of modified macro- and micro-electrode, indicating that the nanoelectrode can not be sufficiently explored in the self-assemble process. Some possible reasons on the poor coverage of nanoelectrode could be suggested. (1) Due to the smaller size of the nanoelectrode, a small probability event occurred on the interaction between mercapto-β-CDs and nanoelectrode; (2) The analytes in solution may react with or further cover, the "bare" portion of the nanoelectrode in some way, thus decreasing the coverage.

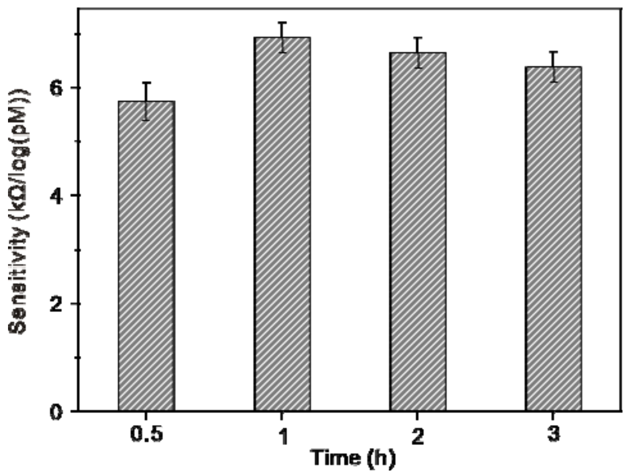

Figure S3. Influence of preconcentration time on sensitivity of PCB-77 at mercapto-β-CD modified 25-μm-diameter gold electrode. Data were evaluated by EIS response toward ultratrace PCB-77 over a concentration ranging from 0 to 32 pM.

It is known that the preconcentration time would affect the amount of gust molecules accumulated onto electrode surface, which was closely associated with the detection limit and sensitivity. In order to achieve the better sensitivity for ultratrace POPs detection at mercapto-β-CD modified electrode, the preconcentration time ranging from 0.5 to 3 h was optimized in solution containing PCB-77 over a concentration range. As depicted in Figure S3, with the increase of preconcentration time from 0.5 to 1 h, the sensitivity of PCB-77 increased and

reached a maximum at 1 h, which was attribute to the increased amount of gust molecules that captured on the modified electrode surface. When the preconcentration time further increased up to 3 h, slight fluctuation without increasing in sensitivity was observed. The results indicated that the an equilibration was reached between gust molecules and host molecules (β-CDs) within 1 h. Therefore, 1 h was chosen as optimized preconcentration time for further analysis.

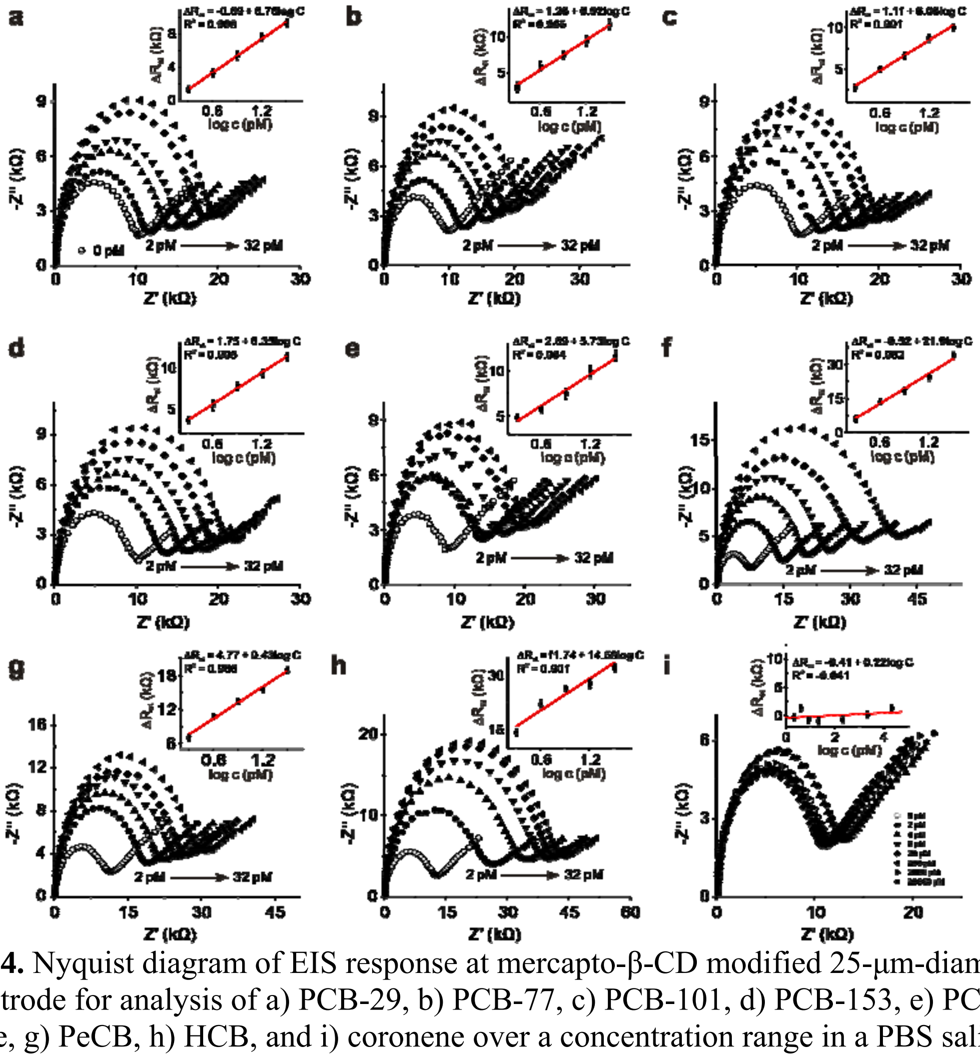

Figure S4. Nyquist diagram of EIS response at mercapto-β-CD modified 25-μm-diameter gold electrode for analysis of a) PCB-29, b) PCB-77, c) PCB-101, d) PCB-153, e) PCB-187, f) lindane, g) PeCB, h) HCB, and i) coronene over a concentration range in a PBS saline solution (pH 7.4). In the insets a-i, the corresponding calibration plots of ΔR_{et} against the logarithmic value of gust molecular concentrations.

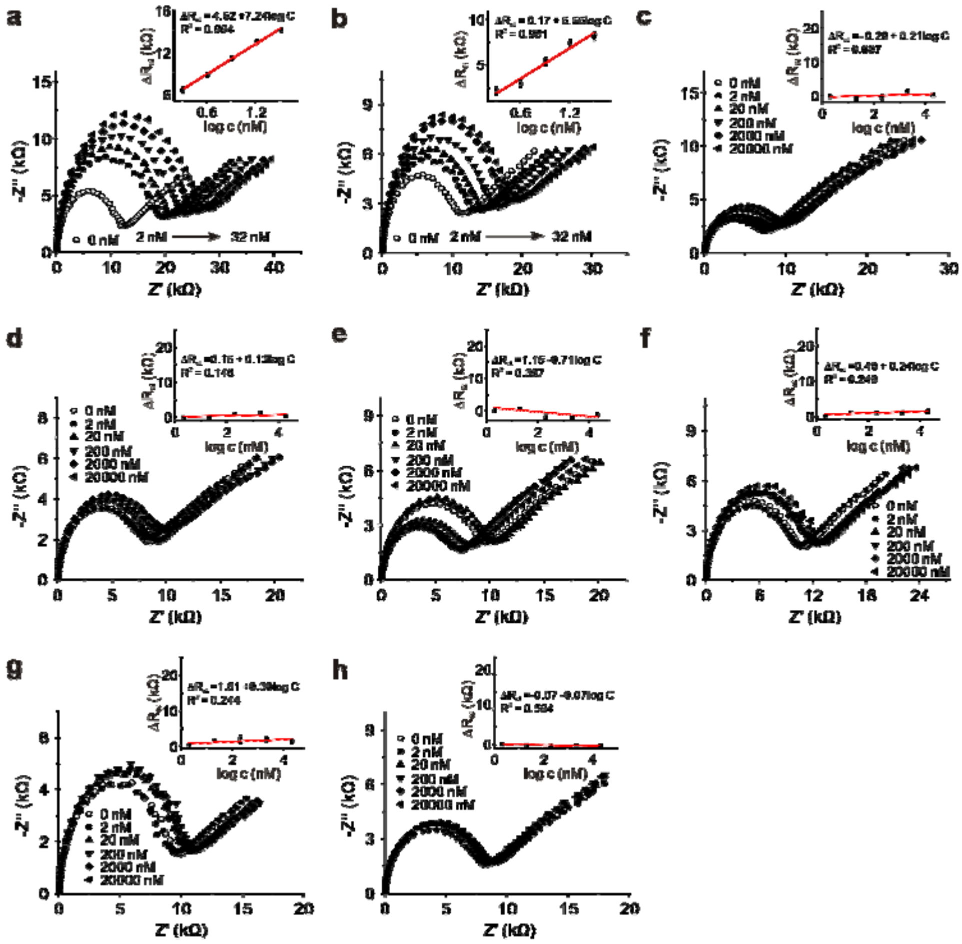

Figure S5. Nyquist diagram of EIS response at mercapto-β-CD modified 25-µm-diameter gold electrode for analysis of a) As(III), b) As(V), c) Cd(II), d) Cu(II), e) Hg(II), f) Pb(II), g) Zn(II), and h) Mn(II) over a concentration range in a PBS saline solution (pH 7.4). In the insets a-h, the corresponding calibration plots of ΔR_{et} against the logarithmic value of gust molecular concentrations, respectively.

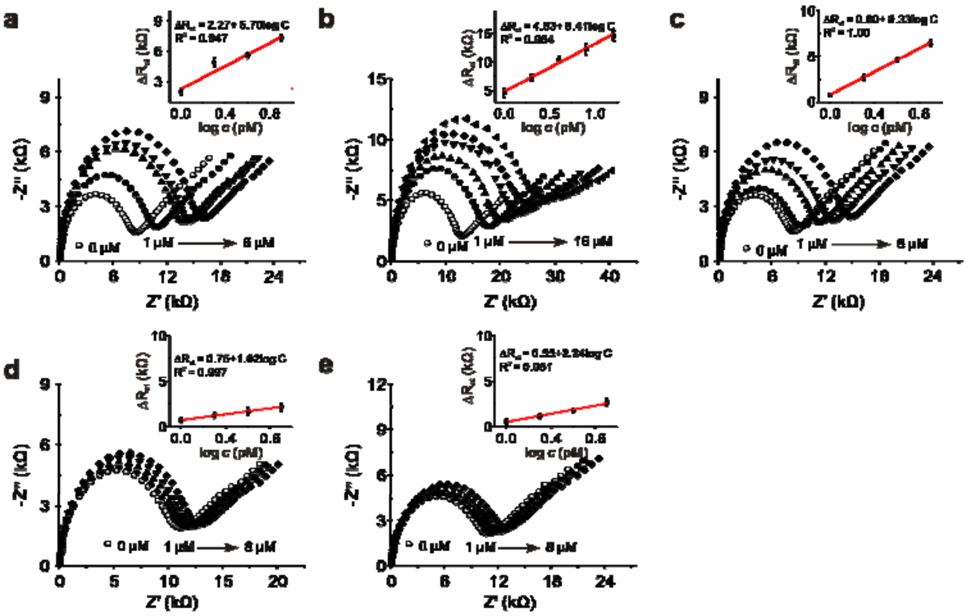

Figure S6. Analysis of bivalent metal ions in alkaline solution. Nyquist diagram of EIS response at mercapto-β-CD modified 25-μm-diameter gold electrode for analysis of a) Cd(II), b) Cu(II), c) Pb(II), d) Zn(II), and e) Mn(II) in alkaline solution (NaOH, pH 11.0) over a concentration range. In the insets a-e), the corresponding linear calibration plot of ΔR_{et} against the logarithmic value of gust molecular concentrations, respectively.

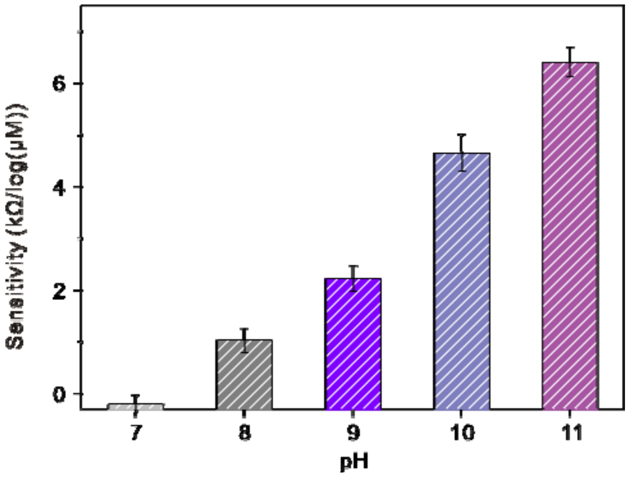

Figure S7. Effect of pH value on sensitivity of Pb(II) at the mercapto-β-CD modified 25-μm-diameter gold electrode. Data were evaluated by EIS of Pb(II) over a concentration ranging from 1 to 8 μM.

Figure S7 shows the change in the sensitivity of Pb(II) at mercapto-β-CD modified 25-μm-diameter gold electrode over different pH values ranging from 7.0 to 11.0. As observed, the sensitivity for Pb(II) continually increased as pH values changed from 7.0 to 11.0, eventually reaching a maximum at pH 11.0. This was probably due to the case, in which with an increase of pH, more hydroxyl groups of β-CD were deprotonated for participating in the formation of Pb(II)−β-CD complexes. Just as reported, Pb(II)–β-CD can be formed with β-

CD anions at pH 9.7−11.5.[4] On the basis of the results, the alkaline solution with pH 11.0 can be selected for impedimetric analysis of Pb(II) and the other bivalent metal ions

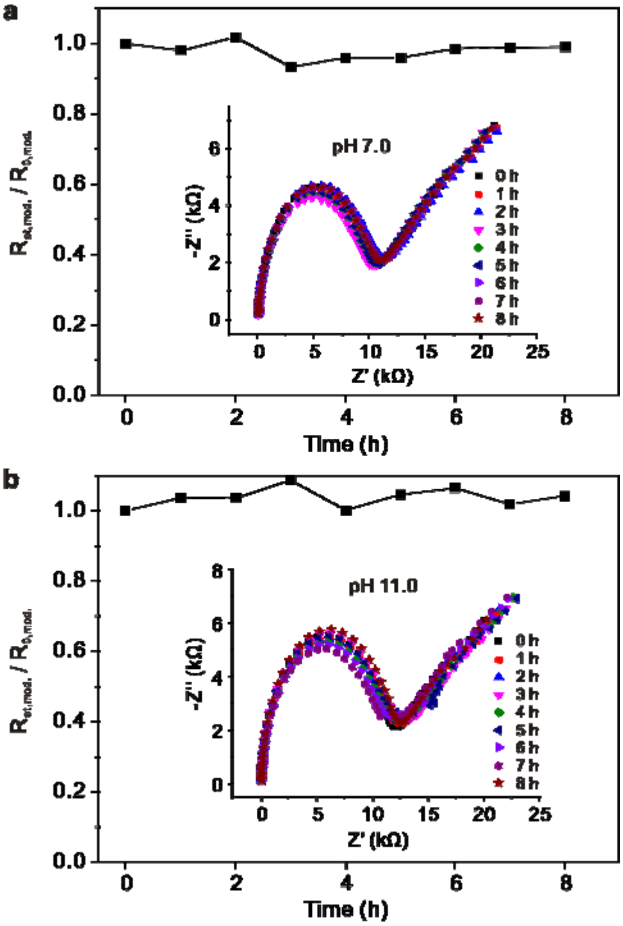

Figure S8. Stability of mercapto-β-CD modified 25-μm-diameter gold electrode in NaOH solution with a) pH=7.0 and b) pH=11.0 monitored by electrochemical impedance. In the insets a,b), dependency of the immerging time on the corresponding EIS response, respectively.

The stability of mercapto-β-CD modified 25-μm-diameter gold electrode was examined to check out its efficiency. Figure S8 shows the EIS response of freshly prepared mercapto-β-CD modified gold electrode after immersing in solutions of pH=7 and pH=11 over a different time, in which the immersion-detection cycling were carried out with repetitive intervals as 1 h. As shown, no obvious changes occurred before and after immersing in solutions (pH 7.0, 11.0) even up to 8 h. The corresponding relative standard deviations (RSDs) were 2.61% (pH 7) and 2.71% (pH 11.0), respectively. The results demonstrated that the mercapto-β-CD

modified 25-μm-diameter gold electrode exhibited robust stability in neutral and medium basic solution.

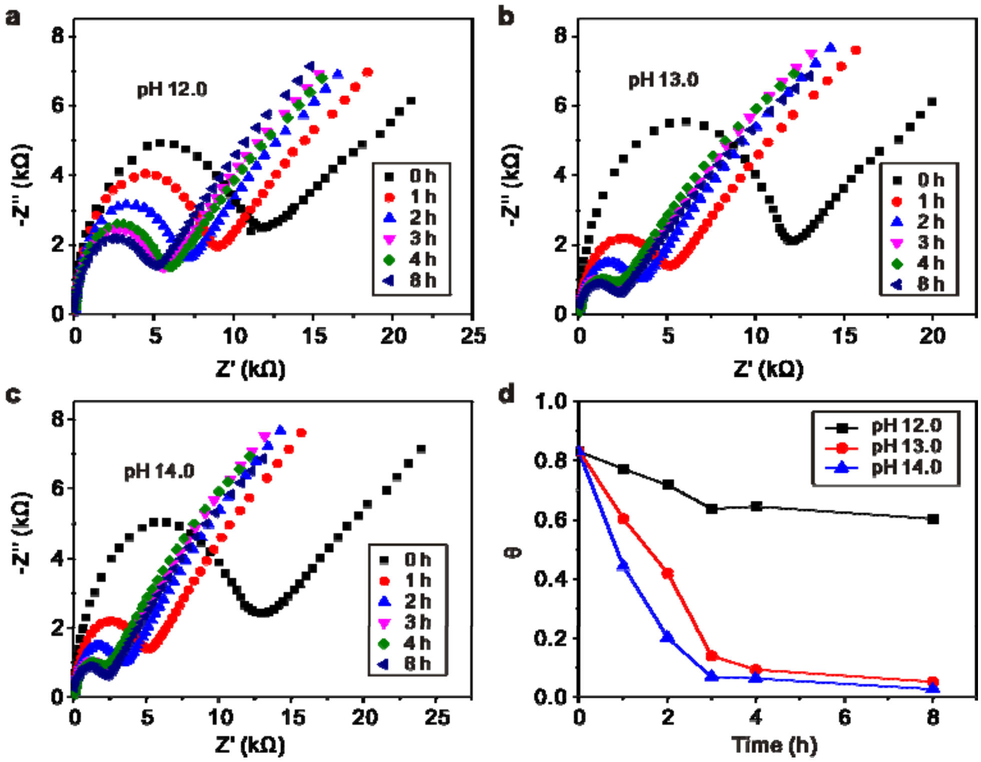

Figure S9. Dependency of immerging time on EIS response at 25-μm-diameter gold electrode in NaOH solution with a) pH=12, b) pH=13, c) pH=14. d) Dependency of immerging time on the corresponding surface coverage θ at 25-μm-diameter gold electrode.

Besides, the strongly alkaline condition was under consideration. The impedance behavior of mercapto-β-CD modified 25-μm-diameter gold electrode was evaluated in a pH range from 12.0 to 14.0 (Figure S9). As shown, R_{et} in strongly alkaline conditions was continually decreased with increasing the immerging time. Then R_{et} tended to level off when the immerging time exceeded 4 h. The surface coverages were decreased by 22.31%, 89.37%, 92.28% after immerging 4 h in solutions with pH 12, 13, 14, respectively. In strongly alkaline solutions (pH 13, 14), the significant decrease was found and R_{et} was almost similar to the bare gold electrode (~ 200 Ω) (Figure S9c and d). We suggested that the thiolated SAMs chemisorpted on gold electrode surface may undergo desorption by breaking Au-S bond in strongly alkaline solutions.[5] Hence, considering the stability of mercapto-β-CD modified

electrode, it was not suitable for application in strongly alkaline solutions. Alternatively, the pH value of the test solution should be adjusted less than 12 before each electroanalysis.

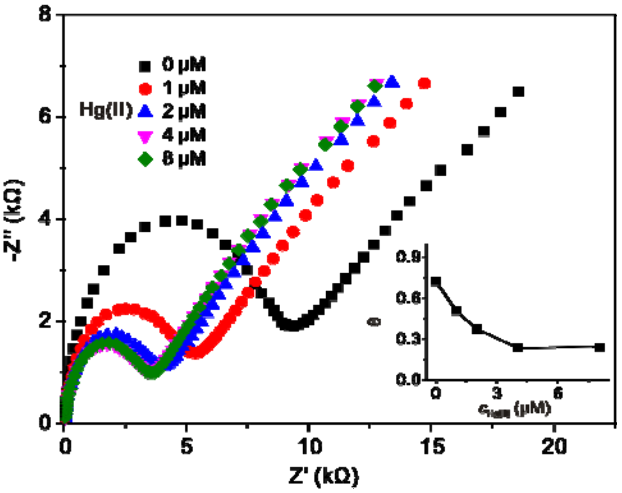

Figure S10. Nyquist diagram of EIS response at mercapto-β-CD modified 25-μm-diameter gold electrode in the presence of Hg(II) in NaOH solution (pH=11). Inset: corresponding surface coverage θ with the addition of Hg(II).

The effect of Hg(II) on the impedance behavior of mercapto-β-CD modified 25-μm-diameter gold electrode was investigated as well. Figure S10 shows the EIS response after a preconcentration of Hg(II) over a different concentration (from 0 to 8μM) in solution (pH 11.0). With the increase of Hg(II), an obvious decrease in R_{et} was observed. The surface coverage θ decreased by 66.23% when the concentration of Hg(II) was up to 8 μM (Figure S10b). It has been reported that Hg(II) was capable of removing the –SH chemisorpted on the gold surface,[6] which would decompose of Au-S bond between gold electrode and mercapto-β-CD. As a result, the presence of Hg(II) could seriously damage of mercapto-β-CD SAMs on gold electrode surface and lead the instability of the modified electrode.

Table S1. Comparison of performance for electrochemical detection of As(III)

Electrode	Electrolyte	Methods	Linearity range (nM)	Sensitivity	LOD (nM)	Ref.
Au macroelectrode	1 M HNO_3	SWASV	2000−20000	0.013 nA nM^{-1}	11.5	[7]
AuNPs-GCE	0.5 M H_2SO_4	LSASV	20−3000	0.202 nA nM^{-1}	0.9	[8]
AuNPs-GCE	1 M HCl	SWASV	0.67−200	0.13 nA nM^{-1}	0.33	[9]
AuNPs-GCE	3 M HCl	LSV	0−1000	0.32 nA nM^{-1}	24	[10]
AuNPs-CNTs	0.1 M HCl	SWV	10−100	1.985 nA nM^{-1}	13.33	[11]
CNTs/AuNPs-GC-GCE	1 M H_2SO_4	LSV	100−5000	0.006 nA nM^{-1}	30	[12]
PtNPs/CNTs	0.1 M H_2SO_4	LSV	10^4−1.5×10^5	0.0007 nA nM^{-1}	1.6	[13]
Au nanofilm GCE	1 M HCl	LSV	10−5000		0.533	[14]
PtNPs-BDD	0.1 M H_2SO_4	LSV	0−1333		6.67	[15]
Silver electrode	0.1 M HNO_3	SWASV	2000−20000	0.0026 nA nM^{-1}	630	[16]
Ir-BDD	0.1 M PBS (pH 4.0)	Amperometry	100−10^5	0.037 nA nM^{-1}	20	[17]
AuNPs-SPE	1 M HCl	LSV	40−400		5.33	[18]
Au-coated BDD	1 M HCl	DPASV	1−533		0.067	[19]
RGO/PbO-GCE	1 M HCl	SWASV	10−10^6	13 µA (log $nM)^{-1}$	10	[20]
mercapto-β-CD-Au microelectrode	neutral solution	EIS	2−32	7.24 kΩ (log $nM)^{-1}$	0.26	this work

Au NPs: gold nanoparticles; GCE: glassy carbon electrode; CNTs: carbon nanotubes; GC: glassy carbon microspheres; PtNPs: platinum nanoparticles; BDD: boron-doped diamond; SPE: screen-printed electrode; RGO: graphene oxide; PBS: phosphate buffer solution; SWASV: square wave anodic stripping voltammetry; SWV: square wave voltammetry; LSASV: linear sweep anodic stripping voltammetry; DPASV: differential pulse anodic stripping voltammetry; LSV: linear sweep voltammetry.

Table S2. Comparison of performance for electrochemical detection of Cr(VI)

Electrode	Electrolyte	Methods	Linearity range (nM)	Sensitivity	LOD (nM)	Ref.
AuNPs-GCE	0.1 M HCl	SWV	3−900	6 nA nM^{-1}	0.19	[21]
CTAB-CPE	0.3 M HCl	DPCSV	500−50000		50	[22]
Ir-based mercury electrode	acetate buffer (pH 5.6)	ASV	20−8000	0.4 nA nM^{-1}	9.6	[23]
Ti/TiO_2NT-Au electrode	0.1 M HCl	Amperometry	100–10^5	7 nA nM^{-1}	30	[24]
bismuth film electrode	acetate buffer (pH 4.5)	ASV	200−1000	10 nA nM^{-1}	101.3	[25]
poly-L-histidine-SPCE	acetate buffer (pH 4.0)	LSV	100–1.5×10^5	1.1 nA nM^{-1}	46	[26]
Au-SPE	0.05 M H_2SO_4	LSV	10^4–1.6×10^6	0.0541 nA nM^{-1}	4400	[27]
pyridine-functionalized-CE	0.1 M HCl	SWV	400−8000	0.113 nA nM^{-1}	88	[28]
graphite screen printed electrode	0.1 M H_2SO_4	LSV	2000−20000	0.041 nA nM^{-1}	365	[29]
AuNPs-Au electrode	0.1 M HCl	Amperometric	4−60	1.56 nA nM^{-1}	1.9	[30]
AgNPs-GCE	0.1 M HNO_3	LSV	40−4000	0.057 nA nM^{-1}	12.8	[31]
Au electrode	0.1 M HCl	LSV	10^5–10^8	0.03 nA nM^{-1}	4300	[32]
Au plated carbon composite electrode	0.3 M HNO_3	LSV	400−40000	0.036 nA nM^{-1}	84	[33]
mercapto-β-CD-Au microelectrode	neutral solution	EIS	2−32	17.5 kΩ $(\log nM)^{-1}$	0.24	this work

CPE: carbon paste electrode; TiO_2NTs: titania nanotubes; SPCE: screen-printed carbon electrode; DPCSV: differential pulse cathodic stripping voltammetry.

Reference

[1] a) M. C. Henstridge, R. G. Compton, *Chem. Rec.* **2012**, *12*, 63; b) X. J. Huang, A. M. O'Mahony, R. G. Compton, *Small* **2009**, *5*, 776.

[2] Y. Li, D. Bergman, B. Zhang, *Anal. Chem.* **2009**, *81*, 5496.

[3] R. Zhang, G.-D. Jin, D. Chen, X.-Y. Hu, *Sens. Actuators B: Chemical* **2009**, *138*, 174.

[4] a) E. Norkus, R. Vaitkus, *Carbohydr. Res.* **2002**, *337*, 1657; b) E. Norkus, G. Grinciene, R. Vaitkus, *Carbohydr. Res.* **2002**, *337*, 1657.

[5] J. Y. Lee, S. M. Park, *J. Phys. Chem. B* **1998**, *102*, 9940.

[6] G. K. Darbha, A. K. Singh, U. S. Rai, E. Yu, H. Yu, P. Chandra Ray, *J. Am. Chem. Soc.* **2008**, *130*, 8038.

[7] A. O. Simm, C. E. Banks, R. G. Compton, *Electroanalysis* **2005**, *17*, 335.

[8] T. Gu, L. Bu, Z. Huang, Y. Liu, Z. Tang, Y. Liu, S. Huang, Q. Xie, S. Yao, X. Tu, X. Luo, S. Luo, *Electrochem. Commun.* **2013**, *33*, 43.

[9] Y. Lan, H. Luo, X. Ren, Y. Wang, L. Wang, *Anal. Lett.* **2012**, *45*, 1184.

[10] M. M. Hossain, M. M. Islam, S. Ferdousi, T. Okajima, T. Ohsaka, *Electroanalysis* **2008**, *20*, 2435.

[11] L. Xiao, G. G. Wildgoose, R. G. Compton, *Anal. Chim. Acta* **2008**, *620*, 44.

[12] X. Dai, G. G. Wildgoose, C. Salter, A. Crossley, R. G. Compton, *Anal. Chem.* **2006**, *78*, 6102.

[13] H. Xu, L. Zeng, S. Xing, Y. Xian, L. Jin, *Electrochem. Commun.* **2008**, *10*, 551.

[14] Y. Diu, W. Zhao, J. J. Xu, H. Y. Chen, *Talanta* **2009**, *79*, 243.

[15] S. Hrapovic, Y. L. Liu, J. H. T. Luong, *Anal. Chem.* **2007**, *79*, 500.

[16] A. O. Simm, C. E. Banks, R. G. Compton, *Electroanalysis* **2005**, *17*, 1727.

[17] T. A. Ivandini, R. Sato, Y. Makide, A. Fujishima, Y. Einaga, *Anal. Chem.* **2006**, *78*, 6291.

[18] M. Khairy, D. K. Kampouris, R. O. Kadara, C. E. Banks, *Electroanalysis* **2010**, *22*, 2496.

[19] Y. Song, G. M. Swain, *Anal. Chim. Acta* **2007**, *593*, 7.

[20] G. K. Ramesha, S. Sampath, *Sens. Actuators B: Chemical* **2011**, *160*, 306.

[21] B. Z. Liu, L. Y. Lu, M. Wang, Y. Q. Zi, *J. Chem. Sci.* **2008**, *120*, 493.

[22] I. Svancara, P. Foret, K. Vytras, *Talanta* **2004**, *64*, 844.

[23] J. Wang, J. Wang, B. Tian, M. Jiang, *Anal. Chem.* **1997**, *69*, 1657.

[24] W. Jin, G. S. Wu, A. C. Chen, *Analyst* **2014**, *139*, 235.

[25] J. Li, J. Zhang, H. Wei, E. Wang, *Analyst* **2009**, *134*, 273.

[26] M. F. Bergamini, D. P. dos Santos, M. V. B. Zanoni, *Sens. Actuators B: Chemical* **2007**, *123*, 902.

[27] J. P. Metters, R. O. Kadara, C. E. Banks, *Analyst* **2012**, *137*, 896.

[28] N. A. Carrington, L. Yong, Z.-L. Xue, *Anal. Chim. Acta* **2006**, *572*, 17.

[29] P. M. Hallam, D. K. Kampouris, R. O. Kadara, C. E. Banks, *Analyst* **2010**, *135*, 1947.

[30] B. K. Jena, C. R. Raj, *Talanta* **2008**, *76*, 161.

[31] S. J. Xing, H. Xu, J. S. Chen, G. Y. Shi, L. T. Jin, *J. Electroanal. Chem.* **2011**, *652*, 60.

[32] C. M. Welch, O. Nekrassova, R. G. Compton, *Talanta* **2005**, *65*, 74.

[33] O. Domínguez-Renedo, L. Ruiz-Espelt, N. García-Astorgano, M. J. Arcos-Martínez, *Talanta* **2008**, *76*, 854.
